# The atherogenic index of plasma (AIP) is a predictor for the severity of coronary artery disease

**DOI:** 10.3389/fcvm.2023.1140215

**Published:** 2023-06-27

**Authors:** Ya Li, Yujia Feng, Shu Li, Yulin Ma, Jiesheng Lin, Jing Wan, Min Zhao

**Affiliations:** ^1^ Department of Cardiology, Zhongnan Hospital of Wuhan University, Wuhan, China; ^2^ Department of Intensive Care Unit, Zhongnan Hospital of Wuhan University, Wuhan, China; ^3^ Department of Cardiology, Hubei Jianghan Oilfield General Hospital, Qianjiang, China; ^4^ Institute of Epidemiology, Helmholtz Zentrum München, German Research Center for Environmental Health (GmbH), Neuherberg, Germany; ^5^ Institute for Medical Information Processing, Biometry, and Epidemiology (IBE), Faculty of Medicine, Pettenkofer School of Public Health, LMU Munich, Munich, Germany; ^6^ Demonstration Center for Experimental Basic Medicine Education, School of Basic Medical Sciences, Wuhan University, Wuhan, China

**Keywords:** atherogenic index of plasma, atherogenic index, lipoprotein combine index, coronary artery disease, biomarker

## Abstract

**Objective:**

Dyslipidemia is a key risk factor for coronary artery disease (CAD). This study aimed to investigate the correlation between the atherogenic index of plasma (AIP) and the severity of CAD.

**Methods:**

2,491 patients were enrolled in this study and analyzed retrospectively, including 665 non-CAD patients as the control group and 1,826 CAD patients. The CAD patients were classified into three subgroups according to tertiles of SYNTAX score (SS). Non-high-density lipoprotein cholesterol (Non-HDL-C) was defined as serum total cholesterol (TC) minus serum high-density lipoprotein cholesterol (Non-HDL-C), atherogenic index (AI) was defined as the ratio of non-HDL-C to HDL-C; AIP was defined as the logarithm of the ratio of the concentration of triglyceride (TG) to HDL-C; lipoprotein combine index (LCI) was defined as the ratio of TC∗TG∗ low-density lipoprotein cholesterol (LDL)to HDL-C; Castelli Risk Index I (CRI I) was defined as the ratio of TC to HDL-C; Castelli Risk Index II (CRI II) was defined as the ratio of LDL-C to HDL-C.

**Results:**

The levels of AIP (*P* < 0.001), AI (*P* < 0.001), and LCI (*P *= 0.013) were higher in the CAD group compared with the non-CAD group. The Spearman correlation analysis showed that AIP (*r* = 0.075, *P* < 0.001), AI (*r* = 0.132, *P* < 0.001), and LCI (*r* = 0.072, *P* = 0.001) were positively correlated with SS. The multivariate logistic regression model showed CRI I (OR: 1.11, 95% CI: 1.03–1.19, *P* = 0.005), CRI II (OR: 1.26, 95% CI: 1.15–1.39, *P* < 0.001), AI (OR: 1.28, 95% CI: 1.17–1.40, *P* < 0.001), AIP (OR: 2.06, 95% CI: 1.38–3.07, *P* < 0.001), and LCI (OR: 1.01, 95% CI: 1.01–1.02, *P* < 0.001) were independent predictors of severity of CAD After adjusting various confounders.

**Conclusion:**

CRI I, CRI II, AIP, AI, and LCI were independent predictors of the severity of CAD, which could be used as a biomarker for the evaluation of the severity of CAD.

## 1. Introduction

Coronary artery disease (CAD) is the most prevalent cardiovascular disorder, imposing a formidable risk to individuals in both developed and developing nations. It has been identified as a foremost cause of mortality worldwide, exerting a substantial burden on global public health (1). Coronary artery disease (CAD) is primarily an atherosclerotic condition influenced by various factors, including inflammation, endothelial dysfunction, and oxidative stress. The risk factors associated with CAD development are well-established. Dyslipidemia, particularly elevated levels of total cholesterol (TC), triglycerides (TG), low-density lipoprotein cholesterol (LDL-C), and decreased high-density lipoprotein cholesterol (HDL-C), has been extensively studied and strongly linked to the onset and progression of CAD. In clinical practice, LDL-C reduction has traditionally been the main focus for lipid-lowering therapy. However, residual cardiovascular risk persists even when LDL-C levels reached recommended targets (2). Therefore, we hypothesized that employing a combination of lipid indices may offer a more comprehensive assessment of lipid status as opposed to relying on a single lipid index.

Recently, composite lipid indices, including the atherogenic index of plasma (AIP, the logarithm of the ratio of TG to HDL-C levels), atherogenic index (AI, non-HDL-C/HDL-C), and lipoprotein combine index (LCI, TC∗TG∗LDL/HDL-C) have emerged as potential predictors of CAD risk, surpassing the predictive value of traditional single lipid parameters (3). AIP has been observed to exhibit a strong association and inverse correlation with the diameter of LDL-C particles, serving as an indirect indicator of small, low-density lipoprotein (sdLDL) levels (4). Thus, AIP may be a predictor of CAD. Studies have indicated a significant association between AIP and obesity (5), highlighting AIP as a novel lipid marker for individuals with Type 2 diabetes and CAD (3). Further, AIP is a strong predictor of CAD in post-menopausal women (6). The SYNTAX score (SS) is used to evaluate the degree of CAD and the severity of coronary stenosis (7). It is an anatomical scoring system based on coronary angiography. In a recent study, AIP emerged not only as a predictive factor for CAD but also exhibited a positive correlation with a high SYNTAX score, indicating its potential as a marker for evaluating CAD severity (8). However, there is limited research available on the correlation between AIP and SS, and the relationship has not been well established. Therefore, the primary objective of this study was to explore the association between AIP and both the incidence and severity of CAD. Additionally, the study aimed to explore the association of AIP with both the incidence and severity of CAD.

## 2. Materials and methods

### 2.1. Study population

A total of 2,491 patients who underwent coronary angiography were enrolled in our study and were admitted into the Department of Cardiology, Zhongnan Hospital, Wuhan University from January 2014 to December 2019. CAD was defined as ≥50% stenosis of at least one epicardial major coronary artery (i.e., >2 mm in diameter) assessed by coronary angiography according to 2012 ACC/AHA guidelines (either stable or unstable) (9). There were 665 cases of non-CAD hospitalized patients in the control group and 1,826 cases in the CAD group.

Exclusion criteria: patients with lipid-affecting drug use history for more than one month before admission and incomplete lipid profile data, previous myocardial infarction, malignant tumors, severe liver, and renal dysfunction, Prior coronary stent implantation or coronary artery bypass grafting, thyroid dysfunction, decompensated heart failure or cardiomyopathy. This study was approved by the Medical Ethics Review Committee of Zhongnan Hospital. The informed consent was obtained from all participants.

### 2.2. Coronary angiography and SYNTAX score group

Coronary angiography was performed by two experienced interventional cardiovascular physicians, and the results of the angiography were evaluated, the disagreements were resolved by the third cardiologist. Coronary angiography via radial or femoral approach according to Judkin’s method. Stenosis of the right coronary artery (RCA), left main coronary artery, anterior descending artery (LAD), and circumflex artery (LCX) was assessed.

Using the SYNTAX Score I to Assess the Severity of CAD. The SS was calculated using the SYNTAX scoring website (https://syntaxscore.org/). CAD patients were dissected into three subgroups according to the tertiles of their SYNTAX score I: mild group (0 ≤ SS < 6.5), moderate group (6.5 ≤ SS ≤ 14), and severe group (>14).

### 2.3. Laboratory measurements and definition

After fasting for more than 10 h, blood samples were taken from the antecubital vein in the early morning. The serum was collected after the blood has spontaneously coagulated and been centrifuged. Serum lipids and high sensitive C-reactive protein (hs-CRP) were measured with an automatic biochemical analyzer (Beckman Coulter, AU5800, USA). Except for dsLDL and hs-CRP, the detection kits, calibrators and quality control products of other plasma lipids are all AU5800 supporting reagents. HDL-C and LDL-C were measured by the homogeneous method (direct method). TC and TG were measured by the enzymatic method. Lp(a) and hs-CRP was measured by the immunoturbidimetric assay (ITA) (the hs-CRP detection kits were from DiaSys Inc., Shanghai, China). sdLDL was mainly measured by the peroxidase method (the detection kits were from Zybio Inc., Chongqing, China). Non-HDL-C is calculated using the following formula:

non-HDL-C=TC-HDL-C

AI, AIP, LCI, remnant lipoprotein cholesterol (RLP-C), Castelli risk index-I (CRI-I), and Castelli risk index-II (CRI-II) were expressed in mmol/L calculated by the following formulas:

(1) $$\mathrm{AI}=\frac{\mathrm{non}\text{-}\mathrm{HDL}\text{-}C}{\mathrm{HDL}\text{-}C}$$

(2) $$\mathrm{AIP}=\log\left(\;\frac{\mathrm{TG}}{\mathrm{HDL}\text{-}C}\right)$$

(3) $$\mathrm{LCI}=\frac{\mathrm{TC}\times \mathrm{TG}\times \mathrm{LDL}}{\mathrm{HDL}\text{-}C}$$

(4) RLP-C=TC-HDL-C-LDL-C

(5) $$\mathrm{Castelli}\;\mathrm{risk}\;\mathrm{index}\text{-}I=\frac{\mathrm{TC}}{\mathrm{HDL}\text{-}C}$$

(6) $$\mathrm{Castelli}\;\mathrm{risk}\;\mathrm{index}\text{-}\mathrm{II}=\frac{\mathrm{LDL}\text{-}C}{\mathrm{HDL}\text{-}C}$$

According to the 2016 Chinese guidelines for the management of dyslipidemia in adults (10), plasma lipids were grouped according to the following criteria: (1) TC: normal: <5.2 mmol/L, marginal high: 5.2–6.2 mmol/L, and high: ≥6.2 mmol/L; (2) TG: normal: <1.7 mmol/L, marginal high: 1.7–2.3 mmol/L, and high: ≥2.3 mmol/L; (3) HDL-C: normal: ≥1.0 mmol/L, low: <1.0 mmol/L; (4) LDL-C: normal: <3.4 mmol/L, marginal high: 3.4–4.1 mmol/L, and high: ≥4.1 mmol/L; (5) Lp(a): normal: <140 mmol/L, and high: ≥140 mmol/L; (6) non-HDL-C: normal: <4.1 mmol/L, and high: ≥4.1 mmol/L; (7) ApoA-1: normal: <1.6 g/L, and high: ≥1.6 g/L; (8) ApoB: normal: <1.3 g/L, and high: ≥1.3 g/L.

### 2.4. Statistical analysis

Statistical analyses were carried out with statistical package for the social sciences version 23.0 software program (IBM Corp, Armonk, New York, USA). Continuous variables were given as mean ± SD (if normal distribution) and were compared using Student’s *t*-test. However, if variables were not normal distribution, expressed as medians (interquartile ranges). The Mann–Whitney *U*-test was used for comparison between two groups, and the Kruskal-Wallis test was used for comparison between multiple groups. The categorical variables were shown as percentages. The *χ*^2^-test was used to compare mean values of the categorical variables between the groups. The Kolmogorov–Smirnov test was used to assess whether the variables were normally distributed. The correlation of lipid indices with the CAD severity (mild, moderate, severe) was calculated with Spearman’s coefficient analysis. The critical correlation coefficient was calculated through calculator websites (https://mathcracker.com/critical-correlation-calculator). The univariate (i.e., unadjusted) and binary multivariate logistic regressions (i.e., adjusted) were used to estimate the associations of plasm lipids, CRI I, CRI II, AI, AIP and LCI with the incidence of CAD (i.e., the occurrence of CAD was used as the dependent variable for univariate and binary multivariate logistic regression analysis). This analysis was performed in the CAD group and the non-CAD group. The univariate and multivariate logistic regressions were used to estimate the associations of plasm lipids, CRI I, CRI II, AI, AIP, and LCI with the severity of CAD (mild, moderate, and severe). Taking the mild CAD group (0 ≤ syntax score <6.5) as the reference group. For variables with parallel line test *P* > 0.05, the multivariate ordinal logistic regression is used, and for variables with parallel line test *P* < 0.05, the disordered multivariate logistic regression is used. All plasma lipid parameters were included in the univariate logistic regression analysis. We included blood lipids as a categorical variable into logistic regression analysis, which can more clearly show the relationship between blood lipids and CAD and its severity. In multivariate logistic regression analysis, we included potential confounding factors into the analysis, mainly those with a *P*-value <0.5 in the baseline characteristics (i.e., age, gender, smoking, diabetes, hypertension, creatinine, uric acid, hs-CRP). The results were evaluated within a 95% confidence interval (CI) and at a significance level of two-sided *P*-value less than 0.05.

## 3. Results

### 3.1. Basic clinical characteristics of the CAD and non-CAD group

Table 1 shows the basic clinical characteristics and biochemical data of CAD and non-CAD patients. The values of CRI I (*P* < 0.001), CRI II (*P* < 0.001), AIP (*P* < 0.001), AI (*P* < 0.001), and LCI (*P* = 0.013) were significantly higher in CAD group than non-CAD group. Patients in the CAD group had higher age, Cr, UA, FBG, and hs-CRP, higher percentage of male patients, smoking, diabetes, and hypertension compared with the non-CAD group. TC, Lp(a), and TG/HDL were higher in CAD patients, while non-CAD patients had lower HDL, ApoA1, and ApoA1/ApoB levels.

**Table 1:** Basic characteristics between Non-CAD and CAD group.

Characteristics	Non-CAD group (*n* = 665)	CAD group (*n* = 1,826)	*P*-value
Age	58.51 ± 11.00	64 (56,70)	<0.001
Gender [male (%)]	353 (22.1%)	1,243 (77.9%)	<0.001
Smoking	138 (17.2%)	666 (82.8%)	<0.001
Diabetes	80 (13.2%)	524 (86.8%)	<0.001
Hypertension	295 (19.1%)	1,250 (80.9%)	<0.001
FBG (mmol/L)	5.16 (4.71,5.71)	5.60 (4.92,6.78)	<0.001
Cr (mmol/L)	70.00 (58.90,81.00)	73.60 (62.55,85.80)	<0.001
UA (mmol/L)	335.84 ± 94.81	342.20 (286.15,410.75)	<0.001
hs-CRP (mg/L)	1.28 (0.63,2.49)	2.24 (1.01,5.76)	<0.001
TC (mmol/L)	4.25 (3.55,4.92)	4.31 (3.81,5.04)	0.022
TG (mmol/L)	1.43 (1.00,2.10)	1.47 (1.05,2.15)	0.090
HDL-C (mmol/L)	1.16 (0.98,1.39)	1.04 (0.89,1.24)	<0.001
LDL-C (mmol/L)	2.62 (2.12,3.26)	2.66 (2.03,3.28)	0.774
ApoA-1 (g/L)	1.24 (1.08,1.42)	1.14 (1.01,1.32)	<0.001
ApoB (g/L)	0.82 (0.67,1.02)	0.85 (0.70,1.02)	0.086
Lp(a) (mmol/L)	109.95 (53.53,210.63)	121.80 (61.20,266.70)	0.002
RLP-C (mmol/L)	0.51 (0.27,0.80)	0.47 (0.25,0.74)	0.074
Non-HDL (mmol/L)	3.16 (2.59,3.82)	3.18 (2.54,3.86)	0.872
ApoA1/ApoB	1.51 (1.19,1.89)	1.37 (1.09,1.69)	<0.001
CRI-I	3.81 (3.11–4.65)	4.02 (3.27–4.92)	<0.001
CRI-II	2.28 (1.76,2.93)	2.47 (1.87,3.17)	<0.001
TG/HDL-C	1.23 (0.80,1.82)	1.41 (0.89,2.23)	<0.001
AI	2.81 (2.10,3.66)	3.02 (2.27,3.92)	<0.001
AIP	0.11 ± 0.29	0.15 (−0.04,0.35)	<0.001
LCI	14.57 (7.56,28.05)	15.94 (8.36,30.48)	0.013

CAD, coronary artery disease; non-CAD, non-coronary artery disease; FBG, fasting blood glucose; Cr, creatinine; UA, uric acid; hs-CRP, hypersensitive C-reactive protein; TC, total cholesterol; TG, triglyceride; HDL-C, high-density lipoprotein cholesterol; LDL-C, low-density lipoprotein cholesterol; ApoA-1, ApolipoproteinA-1; ApoB, ApolipoproteinB; Lp(a), Lipoprotein(a); RLP-C, Remnant lipoprotein cholesterol; non-HDL, non-high-density lipoprotein cholesterol; ApoA1/ApoB, ApolipoproteinA-1/ ApolipoproteinB ratio; CRI-I, Castelli risk index-I; CRI-II, Castelli risk index-II; TG/HDL-C, triglyceride/ high-density lipoprotein cholesterol ratio; AI, atherogenic index; AIP, atherogenic index of plasma; LCI, lipoprotein combine index.

### 3.2. Basic clinical characteristics of CAD patients

Table 2 shows the basic clinical characteristics and biochemical data of patients with mild, moderate and severe CAD. The AIP, AI, and LCI levels were compared among these three subgroups. The results demonstrated that CRI I (*P* < 0.001), CRI II (*P* < 0.001), AIP (*P* < 0.001), AI (*P* < 0.001), and LCI (*P* = 0.007) levels were higher in the moderate and severe group compared with the mild group,. Patients in the severe CAD group were older and had higher rates of diabetes, as well as increased levels of serum CR, UA, TC, LDL-C, ApoB, non-HDL-C, CRI I, CRI II, TG/HDL-C, AI, AIP, LCI was higher than mild CAD group.

**Table 2:** Clinical characteristics of three subgroups for patients with CAD.

Characteristics	Mild (*n* = 376)	Moderate (*n* = 758)	Severe (*n* = 692)	*P*-value
Age	61.70 ± 10.98	62.58 ± 10.06*	65 (57,72)*^,^**	0.002
Gender [male (%)]	239 (63.6)	504 (66.5)	491 (71.0)	0.114
Smoking	131 (34.8)	278 (36.7)	254 (36.7)	0.949
Diabetes	86 (22.9)	197 (26.0)	232 (33.5)*	0.001
Hypertension	247 (65.7)	510 (67.3)	481 (69.5)	0.600
FBG (mmol/L)	5.42 (4.87,6.11)	5.62 (4.93,6.55)	5.63 (4.90,7.52)*	0.009
Cr (mmol/L)	73.80 (65.40,86.30)	72.30 (62.05,83.90)*	75.95 (62.10,90.30)*^,^**	0.010
UA (mmol/L)	336.20 (281.60,397.40)	341.30 (285.45,403.15)	350.20 (296.60,413.00)*^,^**	0.007
hs-CRP (mg/L)	1.66 (0.80,3.15)	2.26 (1.01,5.06)	2.95 (1.23,6.69)*^,^**	<0.001
TC (mmol/L)	4.21 ± 1.06	4.37 ± 1.07*	4.41 (3.49,4.88)*	0.017
TG (mmol/L)	1.43 (1.00,2.15)	1.53 (1.11,2.18)	1.45 (1.04,2.19)	0.180
HDL-C (mmol/L)	1.08 (0.91,1.23)	1.04 (0.89,1.23)*	1.02 (0.88,1.22)*^,^**	0.001
LDL-C (mmol/L)	2.57 ± 0.89	2.74 (2.11,3.28)	2.78 ± 0.95*	0.002
ApoA-1 (g/L)	1.19 (1.06,1.36)	1.16 (1.03,1.34)*	1.11 (0.99,1.28)*^,^**	0.001
ApoB (g/L)	0.81 (0.65,1.00)	0.88 (0.73,1.07)*	0.84 (0.69,1.05)*	0.027
Lp(a) (mmol/L)	116.70 (53.90,257.90)	117.10 (62.10,242.15)	136.70 (63.10,292.20)	0.154
RLP-C (mmol/L)	0.44 (0.21,0.72)	0.49 (0.26,0.75)	0.42 (0.20,0.70)	0.237
Non-HDL (mmol/L)	3.10 ± 1.02	3.23 (2.71,3.89)*	3.15 (2.40,3.77)*	0.001
ApoA1/ApoB	1.51 (1.13,1.87)	1.36 (1.09,1.66)*	1.30 (1.07,1.67)*	<0.001
CRI-I	3.81 (3.03,4.65)	4.03 (3.30,4.92)*	4.22 (3.41,5.03)*	<0.001
CRI-II	2.45 ± 1.04	2.57 ± 0.94*	2.75 ± 1.07*^,^**	<0.001
TG/HDL-C	1.25 (0.83,2.02)	1.43 (0.97,2.23)*	1.44 (0.93,2.24)*	<0.001
AI	2.83 (2.08,3.76)	3.15 (2.39,3.92)*	2.95 (2.27,3.88)*	<0.001
AIP	0.100 (−0.08,0.30)	0.16 (−0.01,0.35)*	0.16 (−0.03,0.35)*	<0.001
LCI	14.43 (7.57,28.74)	18.12 (9.27,32.05)*	15.88 (8.33,29.45)*	0.007

CAD, coronary artery disease; FBG, fasting blood glucose; Cr, creatinine; UA, uric acid; hs-CRP, hypersensitive C-reactive protein; TC, total cholesterol; TG, triglyceride; HDL-C, high-density lipoprotein cholesterol; LDL-C, low-density lipoprotein cholesterol; ApoA-1, ApolipoproteinA-1; ApoB, ApolipoproteinB; Lp(a), Lipoprotein(a); RLP-C, Remnant lipoprotein cholesterol; non-HDL, non-high-density lipoprotein cholesterol; ApoA1/ApoB, ApolipoproteinA-1/ ApolipoproteinB ratio; CRI-I, Castelli risk index-I; CRI-II, Castelli risk index-II; TG/HDL-C, triglyceride/ high-density lipoprotein cholesterol ratio; AI, atherogenic index; AIP, atherogenic index of plasma; LCI, lipoprotein combine index.

* *P* < 0.05 (compared with mild group).

** *P* < 0.05 (compared with moderate group).

### 3.3. Associations of plasm lipids, CRI-I, CRI-II, AI, AIP, and LCI with the incidence of CAD

In univariate logistic regression analysis, ApoA-1, Lp(a), ApoA-1/ApoB, CRI-I, CRI-II, TG/HDL-C, AI, AIP, and LCI were positively correlated with the risk of CAD (all *P* < 0.05), and HDL-C was negatively correlated with with the risk of CAD (*P* < 0.001). After adjusting potential confounding factors (age, gender, smoking, diabetes, hypertension, creatinine, uric acid, hs-CRP), except for TG/HDL-C (*P* = 0.138), other lipid parameters, AI (OR: 1.27, 95% CI:1.15–1.40, *P* < 0.001), AIP (OR: 2.78, 95% CI: 1.82–4.26, *P* < 0.001), LCI (OR: 1.01, 95% CI: 1.01–1.02, *P* < 0.001), CRI-I (OR: 1.10, 95% CI: 1.01–1.20, *P* = 0.037), and CRI II (OR: 1.24, 95% CI: 1.11–1.40, *P* < 0.001) were still positively correlated with the risk of CAD (all *P* < 0.05), and HDL-C was still negatively correlated with the risk of CAD (*P* < 0.001) (Table 3).

**Table 3:** Logistic regression analysis of lipid parameters with the incidence of CAD.

Variables	Unadjusted		Adjusted	
	OR (95%CI)	*P*-value	OR (95%CI)	*P*-value
TC (mmol/L)				
<5.2	1.0	NA	1.0	NA
≥5.2,<6.2	1.01 (0.78–1.29)	0.961	1.06 (0.79–1.43)	0.680
≥6.2	0.79 (0.54–1.15)	0.217	0.84 (0.54–1.33)	0.465
TG (mmol/L)				
<1.7	1.0	NA	1.0	NA
≥1.7,<2.3	1.13 (0.89–1.43)	0.330	1.18 (0.89–1.56)	0.259
≥2.3	1.18 (0.94–1.48)	0.159	1.14 (0.86–1.51)	0.351
HDL-C (mmol/L)				
≥1.0	1.0	NA	1.0	NA
<1.0	1.99 (1.64–2.42)	<0.001	1.61 (1.28–2.02)	<0.001
LDL-C (mmol/L)				
<3.4	1.0	NA	1.0	NA
≥3.4,<4.1	1.03 (0.80–1.33)	0.821	1.06 (0.79–1.42)	0.714
≥4.1	1.11 (0.76–1.63)	0.578	1.20 (0.75–1.92)	0.439
ApoA-1 (g/L)				
<1.6	1.0	NA	1.0	NA
≥1.6	0.46 (0.33–0.66)	<0.001	0.63 (0.43–0.92)	0.016
ApoB (g/L)				
<1.3	1.0	NA	1.0	NA
≥1.3	1.17 (0.79–1.72)	0.427	1.20 (0.79–1.83)	0.386
Lp(a) (mmol/L)				
<140	1.0	NA	1.0	NA
≥140	1.28 (1.06–1.54)	0.011	1.53 (1.23–1.92)	<0.001
Non-HDL (mmol/L)				
<4.1	1.0	NA	1.0	NA
≥4.1	0.98 (0.78–1.23)	0.884	0.97 (0.74–1.23)	0.849
ApoA-1/ApoB	0.63 (0.53–0.75)	<0.001	0.68 (0.56–0.83)	<0.001
CRI-I	1.14 (1.06–1.23)	<0.001	1.10 (1.01–1.20)	0.037
CRI-II	1.27 (1.15–1.40)	<0.001	1.24 (1.11–1.40)	<0.001
TG/HDL-C	1.11 (1.04–1.18)	0.001	1.05 (0.98–1.13)	0.138
RLP-C (mmol/L)	0.93 (0.80–1.08)	0.341	0.85 (0.71–1.03)	0.095
AI	1.29 (1.18–1.40)	<0.001	1.27 (1.15–1.40)	<0.001
AIP	2.75 (1.91–3.94)	<0.001	2.78 (1.82–4.26)	<0.001
LCI	1.01 (1.01–1.02)	<0.001	1.01(1.01–1.02)	<0.001

OR, Odds ratio; AOR, Adjusted odds ratio for age, gender, smoking, diabetes, hypertension, creatinine, uric acid, and hypersensitive C-reactive protein; CAD, coronary artery disease; CI, Confidence intervals; TC, total cholesterol; TG, triglyceride; HDL-C, high-density lipoprotein cholesterol; LDL-C, low-density lipoprotein cholesterol; ApoA-1, ApolipoproteinA-1; ApoB, ApolipoproteinB; Lp(a), Lipoprotein(a); RLP-C, Remnant lipoprotein cholesterol; non-HDL, non-high-density lipoprotein cholesterol; ApoA1/ApoB, ApolipoproteinA-1/ ApolipoproteinB ratio; CRI-I, Castelli risk index-I; CRI-II, Castelli risk index-II; TG/HDL-C, triglyceride/ high-density lipoprotein cholesterol ratio; AI, atherogenic index; AIP, atherogenic index of plasma; LCI, lipoprotein combine index; NA, not applicable.

### 3.4. Correlation between AIP, AI, LCI and CAD severity subgroups

Spearman correlation analysis was performed on AIP, AI, LCI with CAD severity subgroups (mild, moderate, severe). The critical correlation coefficient calculated through calculator websites is 0.046 (*n* = 1,826, two-tailed *α* = 0.05). The results demonstrated that AIP (*r* = 0.075, *P* < 0.001), AI (*r* = 0.132, *P* < 0.001), LCI (*r* = 0.072, *P* = 0.001), CRI I (*r* = 0.132, *P* < 0.001), and CRI II (*r* = 0.128, *P* < 0.001) were positively correlated with SS subgroups.

### 3.5. Associations of plasm lipids, CRI-I, CRI-II, AI, AIP, and LCI with the severity of CAD (mild, moderate, severe)

In univariate logistic regression analysis, ApoA-1, Lp(a), RLP-C, Non-HDL-C, ApoA-1/ApoB, CRI-I, CRI-II, TG/HDL-C, RLP-C, AI, AIP, and LCI were positively correlated with the severity of CAD (all *P* < 0.05), and HDL-C was negatively correlated with the risk of CAD (*P* < 0.001). After adjusting for potential confounding factors (age, gender, smoking, diabetes, hypertension, creatinine, uric acid, hs-CRP), TC marginal high (OR: 1.68, 95% CI: 1.05–2.69, *P* = 0.031) or high (OR: 1.92, 95% CI: 1.25–2.95, *P* = 0.003) was positively correlated with CAD severity. Compared with patients with normal HDL-C, patients with subnormal HDL-C had an increased risk of severe CAD lesions (OR: 1.72, 95% CI: 1.33–2.23, *P* < 0.001). Meanwhile, Lp(a), RLP-C, ApoA1/ApoB, CRI-I (OR: 1.11, 95% CI: 1.03–1.19, *P* = 0.005), CRI II (OR: 1.26, 95% CI: 1.15–1.39, *P* < 0.001), AI (OR: 1.28, 95% CI: 1.17–1.40, *P* < 0.001), AIP (OR: 2.06, 95% CI: 1.38–3.07, *P* < 0.001), and LCI (OR: 1.01, 95% CI: 1.01–1.02, *P* < 0.001) were still positively correlated with the increased risk CAD severity (Table 4).

**Table 4:** Logistic regression analysis of risk factor of SS.

Variables	Unadjusted		Adjusted	
	OR (95%CI)	*P*-value	OR (95%CI)	*P*-value
TC (mmol/L)				
<5.2	1.0	NA	1.0	NA
≥5.2,<6.2	1.22 (0.82–1.81)	0.334	1.68 (1.05–2.69)	0.031
≥6.2	1.37 (0.96–1.95)	0.080	1.92 (1.25–2.95)	0.003
TG (mmol/L)				
<1.7	1.0	NA	1.0	NA
≥1.7,<2.3	1.08 (0.84–1.37)	0.561	1.11 (0.84–1.47)	0.456
≥2.3	1.13 (0.93–1.37)	0.219	1.19 (0.94–1.50)	0.143
HDL-C (mmol/L)				
≥1.0	1.0	NA	1.0	NA
<1.0 (SS moderate vs. mild)	1.69 (1.37–2.09)	<0.001	1.09 (0.86–1.40)	0.468
<1.0 (SS severe vs. mild)	1.95 (1.58–2.42)	<0.001	1.72 (1.33–2.23)	<0.001
LDL-C (mmol/L)				
<3.4	1.0	NA	1.0	NA
≥3.4,<4.1	1.01 (0.70–1.45)	0.960	1.20 (0.77–1.85)	0.420
≥4.1	1.18 (0.86–1.62)	0.299	1.38 (0.94–2.03)	0.100
ApoA1 (g/L)				
<1.6	1.0	NA	1.0	NA
≥1.6	0.64 (0.45–0.91)	0.013	0.84 (0.59–1.20)	0.330
ApoB (g/L)				
<1.3	1.0	NA	1.0	NA
≥1.3	1.35 (0.98–1.87)	0.070	1.43 (1.02–2.00)	0.036
Lp(a) (mmol/L)				
<140	1.0	NA	1.0	NA
≥140	1.24 (1.06–1.46)	0.009	1.30 (1.07–1.57)	0.007
Non-HDL (mmol/L)				
<4.1	1.0	NA	1.0	NA
≥4.1	1.26 (1.03–1.53)	0.023	1.34 (1.06–1.69)	0.292
ApoA1/ApoB	0.61 (0.51–0.72)	<0.001	0.23 (0.54–0.77)	<0.001
CRI-I	1.13 (1.07–1.20)	<0.001	1.11 (1.03–1.19)	0.005
CRI-II	1.28 (1.18–1.38)	<0.001	1.26 (1.15–1.39)	<0.001
TG/HDL-C	1.05 (1.01–1.09)	0.014	1.04 (0.91–1.09)	0.116
RLP-C (mmol/L)	0.87 (0.76–0.99)	0.043	0.82 (0.69–0.98)	0.026
AI	1.26 (1.17–1.36)	<0.001	1.28 (1.17–1.40)	<0.001
AIP	1.68 (1.22–2.31)	<0.001	2.06 (1.38–3.06)	<0.001
LCI	1.01 (1.00–1.01)	<0.001	1.01(1.01–1.02)	<0.001

OR, Odds ratio; AOR, Adjusted odds ratio for age, gender, smoking, diabetes, hypertension, creatinine, uric acid, and hypersensitive C-reactive protein; SS, SYNTAX score; CI, Confidence intervals; TC, total cholesterol; TG, triglyceride; HDL-C, high-density lipoprotein cholesterol; LDL-C, low-density lipoprotein cholesterol; ApoA-1, ApolipoproteinA-1; ApoB, ApolipoproteinB; Lp(a), Lipoprotein(a); RLP-C, Remnant lipoprotein cholesterol; non-HDL, non-high-density lipoprotein cholesterol; ApoA1/ApoB, ApolipoproteinA-1/ ApolipoproteinB ratio; CRI-I, Castelli risk index-I; CRI-II, Castelli risk index-II; TG/HDL-C, triglyceride/ high-density lipoprotein cholesterol ratio; AI, atherogenic index; AIP, atherogenic index of plasma; LCI, lipoprotein combine index; NA, not applicable.

## 4. Discussion

The present study demonstrated that: (1) The AIP, AI, and LCI in CAD patients were considerably greater than that in non-CAD patients; (2) The AIP, AI, and LCI were positively correlated with the severity of CAD and served as independent risk predictors of the incidence and severity of CAD. These results indicated that AIP, AI, and LCI could be used as biomarkers for the evaluation of severity in CAD patients.

Dyslipidemia is a traditional cardiovascular risk factor, it is the key driver for CAD occurrence and progression (11). A previous study has demonstrated that lipid metabolism disorder is characterized by low LDL-C and high TG for most Chinese patients (12). High TG and low HDL levels are and common lipid phenotype that is strongly correlated with cardiovascular disease. It has been well documented that triglyceride-rich lipoproteins (TRL) are involved in atherogenesis by many mechanisms. A previous randomized controlled trial (RCT) demonstrated that elevated RLP-C and TRL are causally associated with low-grade inflammation, whereas elevated LDL-C is not (13). This may be due to the fact that TRL and its remnants can increase the level of cellular adhesion molecules in plasma, thereby promoting the adhesion of leukocytes and monocytes to atherosclerotic lesions, which may trigger an inflammatory response (14, 15). In addition, TRL accumulation in the plasma causes hyperviscosity through fibrinolysis inhibition, and a procoagulant state by amplifying the coagulation cascade through upregulated platelet aggregation, thrombus formation, and enhanced expression of the plasminogen activating inhibitor-1 (16). HDL facilitates cholesterol efflux from lipid-rich macrophages within atherosclerotic plaques in the arterial walls. This efflux is mediated by the interactions of apolipoprotein A-I (apoA-I) with adenosine triphosphate-binding cassette transporter A1 (ABCA1). The cholesterol is then transported to the liver for metabolism or biliary excretion (17). Furthermore, HDL has anti-inflammatory and anti-oxidative effects by regulating endothelial homeostasis and anti-thrombus through attenuation of platelets aggregation and adhesion responses (18).Based on the mechanisms and functions of triglycerides (TG) and high-density lipoprotein cholesterol (HDL-C) in atherosclerosis, the atherogenic index of plasma (AIP), which is the logarithmic transformation of TG/HDL, has been found to be associated with the incidence of CVD. Additionally, AIP has been negatively correlated with LDL particle diameter and can serve as a surrogate marker for small dense LDL (sdLDL) particles, which are also associated with CVD (19). Therefore, it is more reasonable to use AIP to predict CAD risk.

Growing evidence has shown that AIP was strongly associated with numerous cardiovascular risk factors, such as BMI, visceral fat, TG, and glucose (20, 21). AIP was closely related to obesity, hypertension, DM, insulin resistance, and metabolic syndrome (5, 22). Furthermore, it has been suggested that AIP was a reliable indicator in the prediction of atherosclerosis and cardiovascular complications. Cai et al. observed a significant decrease in the atherogenic index of plasma (AIP) in the control group compared to the CAD group (23). Moreover, an observational study showed a strong correlation between the AIP and the severity of CAD (24). In another study, AIP was significantly related to the progression of coronary artery calcification in Korean adults without CVD, the incidence of coronary artery calcification progression increased gradually with rising AIP through 4.2 year-follow-up (25). Furthermore, a study has demonstrated that an increased AIP may predict the complexity of percutaneous coronary intervention in chronic total occlusion patients and additionally may help to improve the therapeutic intervention of chronic total occlusion (26). A previous study, which included 1,347 non-CAD and 2,253 CAD patients, suggested that AIP and SYNTAX scores were positively correlated and both could be used to predict the severity of CAD (8).

In the current study, AIP was elevated in CAD patients compared with non-CAD patients, and AIP was positively correlated with SS, which indicated a positive correlation between AIP and the severity of CAD. The results were consistent with those of previous studies (8). In addition, AI and LCI were found to be significantly higher in CAD than in non-CAD patients in our study, suggesting that these two lipid indices may also be positively associated with CAD severity. AI represents the ratio of all the atherogenic particles (i.e., non-HDL-C) and all the anti-atherosclerosis particles (e.g., HDL-C); this index has been shown to relate broadly to diseases associated with dyslipidemia, including metabolic syndrome, insulin resistance, and CAD (27, 28). LCI is a non-traditional lipid profile and could be used as a risk factor for acute coronary syndrome (29), Oguntola et al. found that LCI correlated with atherosclerotic vascular disease (30). The current study demonstrated a positive correlation between the severity of CAD and three lipid indices. After adjusting various confounders, AI, AIP, and LCI were still independent risk factors for CAD and SS. Moreover, as AI, AIP, and LCI increase, there is a corresponding worsening of coronary artery stenosis. In this study, the utilization of three composite lipid indices aimed to enhance the prediction of both CAD risk and CAD severity, thereby providing a more comprehensive and accurate assessment. On the other hand, this method will be more feasible and more economical in clinical practice.

This study has several important limitations. First, we did not establish the optimal diagnostic threshold values for these three indices. Further investigation in clinical practice is warranted to determine these threshold values. Second, it is important to acknowledge that this study was conducted in a single center and relied on retrospective data. Therefore, the potential influence of selection bias cannot be completely ruled out, and the results may not fully generalize to the broader population. Lastly, it is worth noting that the SS was determined based on the results of coronary angiography, and objective factors such as the spatial resolution of the coronary angiography system and the size of the catheter used could potentially impact the findings of this study.

## 5. Conclusion

The CRI I, CRI II, AI, AIP, and LCI in the CAD group were significantly elevated compared to the non-CAD group, the CRI I, CRI II, AI, AIP, and LCI positively correlated with the CAD severity, which may be used as a noninvasive biomarker for the evaluation of the complexity of CAD in clinical practice. In addition, CRI I, CRI II, AI, AIP, and LCI could serve as independent predictive factors for CAD and CAD severity.

## Data Availability

The raw data supporting the conclusions of this article will be made available by the authors, without undue reservation.
